# Role for neurological and immunological resilience in the pathway of the aging muscle powerpenia: InCHIANTI study longitudinal results

**DOI:** 10.1007/s11357-025-01536-6

**Published:** 2025-01-30

**Authors:** Raffaello Pellegrino, Roberto Paganelli, Angelo Di Iorio, Matteo Candeloro, Stefano Volpato, Stefania Bandinelli, Antimo Moretti, Giovanni Iolascon, Toshiko Tanaka, Luigi Ferrucci

**Affiliations:** 1 Department of Scientific Research, Campus Ludes, Off-Campus Semmelweis University, 6912 Lugano–Pazzallo, Switzerland; 2 Saint Camillus International University of Health and Medical Sciences, Rome, Italy; 3 Department of Innovative Technologies in Medicine & Dentistry, University “G. d’Annunzio” Chieti-Pescara, Viale Abruzzo 322, 66100 Chieti-Pescara, Italy; 4 Longitudinal Studies Section, Translational Gerontology Branch, National Institute On Aging, National Institutes of Health, Baltimore, MD 21224 USA; 5 Department of Medical Sciences, University of Ferrara, 44121 Ferrara, Italy; 6 Azienda USL Toscana Centro, InCHIANTI, Villa Margherita, Primo Piano Viale Michelangelo, 41, 50125 Florence, Italy; 7 Department of Medical and Surgical Specialties and Dentistry, University of Campania “Luigi Vanvitelli”, 80138 Naples, Italy

**Keywords:** Inflammaging, Muscle, Sarcopenia, Powerpenia, Nervous system

## Abstract

The neuromuscular junction shows several degenerative changes with aging, resulting in a reduction of transmission efficacy. These changes, paired with low-grade chronic inflammation, were considered triggers of the aging muscle processes. The main objective of this study is to assess the role of leukocyte count–derived ratios, nerve conduction velocity (NCV), and compound muscle action potential (CMAP) in determining time-dependent reduction in lower limb muscle explosive strength, a condition that has been defined as powerpenia. The InCHIANTI study enrolled a representative sample from the registry lists of two towns in Tuscany, Italy. Baseline data were collected in 1998, with follow-up visits every 3 years. For the purpose of this analysis, we used 1229 subjects and 3814 follow-up assessments. Subjects with lower values of monocyte-to-lymphocyte ratio (ML-ratio) had higher nerve conduction velocity and higher proximal and distal action potential values; moreover, considering the interaction between age for ML-ratio effect, a statistically significant direct association is found with all the electromyography-parameters. Lower limb muscle power shows a gender dimorphism, male subjects having higher values at baseline, but experiencing steeper decline rate during the follow-up, compared to females. Muscle power was inversely associated with ML-ratio, proximal CMAP, distal CMAP, and NCV. Moreover, we found a direct and statistically significant second-order interaction (age for ML-ratio), meaning that at the same age, increasing ML-ratio increases lower limb muscle power. Lastly, also body composition variation across aging is directly associated with lower limb muscle power. Reduced immunological and neurological homeostasis affects the powerpenia phenotype in a large representative sample of Italian men and women.

**Supplementary Information:**

The online version contains supplementary material available at 10.1007/s11357-025-01536-6.

## Introduction

Muscles are one of the most abundant tissues in the human body, accounting for about 40% of the total weight. During adult life, muscles undergo repair and remodeling processes that are closely related to damage caused by normal daily activity and are aimed to maintain normal function [1]. After the age of 30–40 years, a gradual decline in the regenerative capacity of muscles can be observed [2], which ultimately leads to the clinical manifestation of sarcopenia. The definition of sarcopenia includes a reduction in muscle mass and a decrease in muscle strength, and, according to the European Working Group on Sarcopenia in Older People (EWGSOP), also encompasses a decline in walking speed performance [3]. More recently, muscle power, defined as the “product of the force of contraction and velocity of movement” [4] during an exercise or in the routinely every day activities, was demonstrated to be superior to strength in determining functional status, and in the prediction of functional dependency [5]. Therefore, the theoretical conceptualization of aging muscle includes a qualitative and functional aspect called “muscle failure,” in addition to the mere reduction in muscle size [6]. Freitas et al. had recently proposed the term powerpenia to describe the age-related process that induces a “reduction” in the lower limb power [7], in contrast to the definition of dynapenia, conceptually considered as loss of skeletal muscle strength and/or power [8]. These differences cannot be viewed only as semantic or taxonomic, they also have practical consequences, for example in the design of tailored exercise interventions [5], and eventually in different pharmacological approaches.

Muscle strength is determined by a combination of neurological and muscular components. The neuromuscular junction (NMJ) represents the functional interaction between these two systems, and it has been proposed as one of the determinants of sarcopenia [9]. NMJ is a chemical synapse that transfers nerve action potentials from presynaptic motoneurons to postsynaptic muscle fibers, and activates muscle contractions [6, 10]. With advancing age, NMJ structure shows several degenerative changes such as axonal remodeling, loss of junctional folds, reduced numbers of presynaptic acetylcholine vesicles, and a reduced number of receptors for nicotinic acetylcholine receptors [10]. The denervation process involves preferentially fast muscle fibers (type II) with axonal reinnervation through collateral sprouting of nearby surviving motor axons or motor end plates of slow fibers (type I) [11]. All such morphological alterations induce a reduction in transmission efficacy of the NMJ and impair myofiber excitability.

Surface electromyography (EMG) through electrical stimulation of a peripheral nerve provides a measure of nerve physiology [12]. Two main parameters derived from this examination are nerve conduction velocity (NCV) and compound muscle action potential (CMAP) [12]. Impaired NCV is related to alterations in the myelin physiology [13], while CMAP amplitude is directly related to the number of axons that conduct impulses to the muscle. In the InCHIANTI study, it was demonstrated that intrinsic changes in muscle fibers and the structural organization of muscle tissue that occur with aging correlate with a progressive degeneration of peripheral nerves, specifically with lower CMAP but not NCV [14]. Furthermore, in the same population study, chronic low-grade age-related inflammation (inflammaging) and inadequate antioxidant defenses are associated with an accelerated decline of NCV over the aging process [15].

Recently Ahuja et al. proposed the term “immune resilience” to explain why some individuals remain healthier than others throughout the stressors of life. They hypothesized that this advantage is related to an “optimal immune resilience” (IR) [16]. IR was defined as the capacity to preserve and/or rapidly restore immune functions after an acute challenge, which could also be seen as enhanced immunocompetence [17, 18]. The monocyte-to-lymphocyte ratio (ML-ratio) and the neutrophil-to-lymphocyte ratio (NL-ratio) are considered proxy measures of (im)-balance between innate and adaptive immunity. These indices are derived from leukocyte counts in circulation and were proposed as markers of inflammation [19] and as prognostic factors and predictors of cancer [20], dementia [21], and cardiovascular diseases [22]. The NL-ratio and ML-ratio were proposed as reliable markers of biological age, predicting mortality risk and comorbidities in aging individuals [22], suggesting this ratio is a proxy of immunosenescence and inflammaging [22]. Immunosenescence has been considered as part of the pathway that links aging with the reduction of muscle quality and strength [23]. Interestingly, mononuclear phagocytes, which belong to the innate immune system and encompass monocytes, macrophages, and dendritic cells, express nicotinic acetylcholine receptors similarly to the neuromuscular junction [24]. Those cells play pivotal roles in host defense against infection but also in several debilitating diseases that are characterized by inflammation [24], and in age-dependent chronic low-grade inflammation [25]. Mononuclear phagocytes can both stimulate and control inflammation, and they have the capacity to promote nerve repair [26].

Our hypothesis considers that age-related changes in the ratios of leukocyte cell absolute count may not only express a general inflammatory state, but also represent more widespread and organic damage. These changes in the ratios represent an immunological phenotypic response to aging that may influence the physiology of the NMJ and consequently represent one of the determinants of the age-related muscle power reduction (powerpenia) [7].

The main objective of this study, conducted in a large and representative sample of Italian men and women, is to assess NL-ratio, ML-ratio, NCV, and CMAP correlate with time-dependent variations in lower limb muscle power over a 12-year period.

## Methods

InCHIANTI is a cohort study looking at determinants that contribute to the decline of mobility in aging people. The InCHIANTI study is a representative sample for age and sex of the free-living Italian population. The participants were all Europeans of Caucasian origin. The study design and data collection have been previously described elsewhere [27].

### Electromyography

Measurement of nerve motor conduction velocity was performed on the right superficial peroneal nerve using a standard neurophysiologic equipment (ENMG Myto, EBNeuro, ESAOTE, Florence, Italy). The temperature of the testing room was kept at 26–27 °C so that the testing leg is maintained at physiologic temperature. The measurements were obtained while dorsal foot skin temperature was between 30 and 34 °C. If necessary, the skin over the muscle was warmed up with an infrared lamp. The details of the EMG execution protocol in the InCHIANTI study were largely described elsewhere [14, 15], and in Supplementary Material Fig. 1. The following parameters of nerve conduction studies were measured: (a) the proximal and distal amplitude of the compound muscle action potential (CMAP); (b) nerve conduction velocity (NCV) [12].

### Lower limb muscle power evaluation

Maximal voluntary leg extension power was evaluated with the Nottingham power rig (Medical Engineering Unit, University of Nottingham Medical School, Nottingham, UK) [28] and in Supplementary Material Fig. 2, according to the leg extension power rig user manual.

The participants were seated in an upright position, arms folded across the chest, and knees flexed with one foot placed on the floor and the other foot placed on the dynamometer pedal. The sitting position was determined so that the knee reached 15° of flexion (0° is full extension) at the end of the footplate movement [2, 28]. Participants familiarized with the procedure in two warm-up trials and then instructed to push the pedal forward as hard and fast as possible. Measurements were repeated for each limb until maximal power could not be increased further. The highest lower limb muscle power was selected for further analysis [2].

### Laboratory assay

Differential white blood cell count was assessed through an automated system at the Laboratory SS. Annunziata Hospital, Azienda Sanitaria-10, Florence, Italy, using at baseline a Hematology SE 9000 Autoanalyzer (Sysmex, Kobe, Japan, provided by DASIT, Milan, Italy); at follow-up 1, a Coulter LH 750 Hematology Autoanalyzer (Beckman Coulter Inc, Brea, CA, USA); and at follow-up 2 and follow-up 3, a Sysmex XE 2100 (DASIT – Milan, Italy). Neutrophil-to-lymphocyte-ratio (NL-ratio) and monocyte-to-lymphocyte ratio (ML-ratio) were derived from leukocyte absolute number count [19]. Serum high-sensitivity C-reactive protein (HS-CRP) was measured by immunonephelometric assay and monoclonal antibodies in duplicate with the Dade Behring BN II nephelometer (Dade Behring Inc., Deerfield, IL).

### Diseases and comorbidities

The diagnosis of major medical conditions was ascertained according to pre-established criteria that combine clinical record and general practitioner report, and supported by physical examination, blood tests, and drug prescription [29]. The multimorbidity score was calculated summing the number of diseases reported at baseline and all follow-up visits (angina, cancer, hepatic diseases, acute myocardial infarction, congestive heart failure, stroke, Parkinson disease, peripheric arterial disease, diabetes, COPD, asthma, and osteoarthritis).

### Tibial peripheric quantitative computed tomography

Peripheral quantitative computed tomography (pQCT) was performed by the XCT 2000 device (Stratec Medizintechnik, Pforzheim, Germany) [30]. The images obtained from the pQCT were analyzed using the BonAlyse software (BonAlyse Oy, Jyvaskyla, Finland) [31].

The following parameters were derived from the pQCT images measured at 38% tibia length [32]: calf muscle cross-sectional area (cm^2^), fat cross-sectional area (cm^2^), and muscle density (mg/cm^3^); all the estimates are measured from a transverse scan performed at 38% of the tibia length from the distal tip of the tibia.

### Body composition

Body mass index (BMI, kg/m^2^) was calculated using objectively measured height and weight. Weight was measured to the nearest 0.1 kg using a high-precision mechanical scale and standing height to the nearest 0.1 cm with a wall measure with participants wearing light indoor clothes and no shoes [27].

### Statistical analysis

Descriptive data are shown as mean ± standard error, and as absolute number and percentages, for continuous and categorical variables, respectively, according to times of the study. For continuous variables, linear mixed models with random intercept and random slope were applied to assess follow-up differences, whereas for trend, the chi-square test was applied for dichotomous variables.

Linear mixed models were used to investigate the potential associations between white cell count (neutrophil, monocyte, and lymphocyte) ratios with ENMG markers, using time since baseline as the time scale. Age, sex, multimorbidity scores, body mass index, and HS-CRP were considered as confounders; second-order interactions were explored and reported if statistically significant.

Longitudinal linear mixed models were applied using time since baseline as the time scale to identify factors associated with change over time of lower limb power. Three different models were considered according to variation in ENMG markers (proximal CMAP, model A; distal CMAP, model B; nerve conduction velocity, model C); age, sex, multimorbidity scores, body mass index, and HS-CRP were considered as confounders; and second-order interaction was explored and reported if statistically significant.

SAS version 9.4 for Windows (SAS Institute, Inc., Cary, NC) was used for all data processing and statistical analyses. We set the level of statistical significance at *p* < 0.05 (two-sided).

## Results

The main characteristics of the population enrolled in the study, both at baseline and at subsequent follow-up times, are reported in Table 1. In the study, 1229 subjects were enrolled, and there was a total of 3814 assessments. Chronological age represents the population’s mean age at the specific follow-up, whereas age at baseline represents the mean age at the enrollment in the study, of those subjects who were alive in the specific follow-up. During the study times, mean chronological age, comorbidities, NL-ratio, ML-ratio, monocyte number, lymphocyte number, fat area, muscle area, and density increased, while lower limb power strength, distal and proximal CMAP, and NCV decreased (Table 1).

**Table 1:** Descriptive of the InCHIANTI study population according to time of the study

	Baseline	Follow-up 1	Follow-up 2	Follow-up 3	*p*-value
	1229	990	890	705	
Age	68.12 ± 15.28	66.65 ± 15.34	64.97 ± 15.39	63.41 ± 15.24	< 0.001
Chronological age	68.12 ± 15.28	69.69 ± 15.34	71.05 ± 15.39	72.54 ± 15.26	< 0.001
Sex female	648 (54.96)	478 (53.77)	409 (52.98)	311 (51.83)	0.76
Body mass index	27.18 ± 4.13	26.25 ± 4.04	26.90 ± 4.22	27.08 ± 4.25	0.97
Multimorbidity	1.31 ± 1.38	1.79 ± 1.63	2.07 ± 1.74	2.45 ± 1.81	< 0.001
NL-ratio	2.14 ± 0.98	1.99 ± 0.86	2.19 ± 1.20	2.15 ± 1.20	0.004
ML-ratio	0.18 ± 0.08	0.21 ± 0.08	0.28 ± 0.12	0.28 ± 0.14	< 0.001
C-reactive protein	4.63 ± 8.62	3.87 ± 8.61	4.54 ± 8.85	4.15 ± 8.30	0.74
Muscle area^a^	36.78 ± 8.44	38.17 ± 8.61	36.56 ± 8.51	36.59 ± 9.61	< 0.001
Muscle density^a^	72.62 ± 3.74	72.04 ± 3.59	72.88 ± 3.48	73.02 ± 3.67	< 0.001
Fat area^a^	11.92 ± 7.80	12.08 ± 8.41	12.71 ± 7.96	13.50 ± 8.53	< 0.001
Lower limb power (W)	124.68 ± 81.39	149.36 ± 81.57	129.10 ± 79.46	112.74 ± 67.54	< 0.001
Monocytes (*n*, K/µL)	0.32 ± 0.12	0.38 ± 0.14	0.48 ± 0.16	0.50 ± 0.16	< 0.001
Neutrophils (*n*, K/µL)	3.74 ± 1.26	3.58 ± 1.14	3.66 ± 1.24	3.69 ± 1.31	0.87
Lymphocytes (*n*, K/µL)	1.91 ± 0.65	1.96 ± 0.64	1.87 ± 0.69	1.94 ± 0.76	0.08
Distal CMAP (mV)	6.67 ± 3.61	4.28 ± 3.00	4.55 ± 2.96	4.71 ± 3.18	< 0.001
Proximal CMAP (mV)	6.08 ± 3.13	4.00 ± 2.93	3.85 ± 2.73	4.13 ± 2.63	< 0.001
NCV (m/s)	44.62 ± 3.93	43.99 ± 4.92	40.62 ± 4.06	44.15 ± 5.23	< 0.001

^a^Estimates are measured from a transverse scan performed at 38% of the tibia length from the distal tip of the tibia; calf muscle cross-sectional area (cm^2^), fat cross-sectional area (cm^2^), muscle density (mg/cm^3^)

### Longitudinal analysis

#### Distal CMAP of peroneus

In Table 2, the Linear Mixed Model analysis for the variation through times of peroneus distal CMAP is reported. The unconditional means model provides insights into the distal CMAP, independent of individuals and time. The distal CMAP mean value was found to be 5.26 ± 0.07 (*p*-value < 0.001), with a variance within subjects of 8.72 ± 0.27 (*p*-value < 0.001) and a variance between subjects of 2.97 ± 0.27 (*p*-value < 0.001). This indicates that 75% of the total variation in the EMG test can be attributed to differences between subjects (Table 2, model A). Moving to the unconditional growth model (Table 2, model B), the age effect was assessed, and it was estimated that the distal CMAP mean decreases by − 0.08 ± 0.01 (*p* < 0.001) for every year of age. Furthermore, a gender dimorphism was observed, with males exhibiting a higher distal CMAP mean (*β* ± S.E, 3.09 ± 0.61; *p*-value < 0.001), and they lose a higher amount of CMAP during the aging process (interaction age for sex, *β* ± S.E γ _10*01_, − 0.04 ± 0.01; *p*-value < 0.001), compared to females (Table 2, model C). In the fully adjusted model D, the role of NL-ratio was assessed, whereas in model E, the role of ML-ratio was assessed. Both models were adjusted for age, sex, their interaction, body mass index, multimorbidity score, and markers of low-grade chronic inflammation. Low ML-ratio levels were associated with a higher mean CMAP value (*β* ± S.E, − 11.36 ± 3.08; *p*-value < 0.001, Table 2, model E), independently from all the potential confounders considered. Moreover, a statistically significant interaction between chronological age and ML-ratio (*β* ± S.E γ _10*02_, 0.10 ± 0.04; *p*-value = 0.01) could be reported, meaning that within subjects with the same age, higher ML-ratio levels are directly associated with a larger amplitude of the peroneus-superficialis distal CMAP. On the contrary, NL-ratio levels were directly associated with distal CMAP and when was considered the second-order effect an indirect association could be found (Table 2, model D; *β* ± S.E, − 0.01 ± 0.004; *p*-value = 0.03). In both models, age, sex, their interaction, and multimorbidity were also statistically significantly associated with distal CMAP.

**Table 2:** Linear mixed model, factors associated to variation of peroneus distal CMAP according to times of the study (EMG)

			Model A Unconditional means model	Model B Unconditional growth model	Model C Interaction model	Model D Fully adjusted NL-ratio	Model E Fully adjusted ML-ratio
Initial status	Intercept	γ _00_	5.26 ± 0.07***	10.90 ± 0.31***	9.38 ± 0.42***	6.95 ± 0.83***	10.22 ± 0.79***
	Sex (males)	γ _01_			3.09 ± 0.61***	3.05 ± 0.62***	3.12 ± 0.61***
	NL-ratio	γ _02 NLr_				0.70 ± 0.32*	
	ML-ratio	γ _02 MLr_					− 11.36 ± 3.08***
	BMI	γ _03_				0.03 ± 0.02	0.03 ± 0.02
	Multimorbidity	γ _04_				− 0.31 ± 0.04***	− 0.28 ± 0.04***
	CRP	γ _05_				0.01 ± 0.01	0.01 ± 0.01
Rate of change	^&^	γ _10_		− 0.08 ± 0.01***	− 0.07 ± 0.01***	− 0.03 ± 0.01**	− 0.07 ± 0.01***
	Interaction	γ _10*01_			− 0.04 ± 0.01***	− 0.04 ± 0.01***	− 0.03 ± 0.01***
	Interaction	γ _10*02_				− 0.01 ± 0.004*	
	Interaction	γ _10*02_					0.10 ± 0.04*
Level 1	Within person	δ ^2^_e_	8.72 ± 0.27***	7.70 ± 0.30***	7.70 ± 0.30***	7.59 ± 0.29***	7.56 ± 0.23***
Level 2	In initial status	δ ^2^_0_	2.97 ± 0.27***	4.59 ± 0.93***	4.38 ± 0.93***	3.94 ± 0.90***	3.66 ± 0.52***
	In rate of change	δ ^2^_1_		0.17 ± 0.12	0.17 ± 0.12	0.09 ± 0.11	0.07 ± 0.11
	Covariance	δ _01_		− 0.67 ± 0.31*	− 0.66 ± 0.31*	− 0.46 ± 0.30	− 0.29 ± 0.10**
		AIC	17,104	16,750	16,719	16,667	16,617

*p < 0.05; **p < 0.01; ***p < 0.001

#### Proximal CMAP of peroneus

In Table 3, the linear mixed model analysis for the variation through times of peroneus-superficialis proximal CMAP is reported. The unconditional means model provides insights into the proximal CMAP, independent of individuals and time. The proximal CMAP mean value was found to be 4.73 ± 0.07 (*p*-value < 0.001), with a variance within subjects of 6.94 ± 0.21 (*p*-value < 0.001) and a variance between subjects of 2.54 ± 0.22 (*p*-value < 0.001). This indicates that 73% of the total variation in the test can be attributed to differences between subjects (Table 3, model A). Moving to the unconditional growth model (Table 3, model B), the age effect was assessed, and it was estimated that the proximal CMAP mean decreases by − 0.08 ± 0.01 (*p* < 0.001) for every year of age. Furthermore, a gender dimorphism was observed, with males exhibiting a higher proximal CMAP mean of 2.62 ± 0.55 (*p*-value < 0.001) and losing more CMAP during the aging process (interaction age for sex, γ _10*01_, − 0.03 ± 0.01; *p*-value < 0.001), compared to females (Table 3, model C). Finally, in the fully adjusted model D, the role of NL-ratio, and in model E, the role of ML-ratio were assessed. Both models were adjusted for age, sex, their interaction, body mass index, multimorbidity score, and markers of low-grade chronic inflammation (CRP). The ML-ratio levels were indirectly associated with proximal CMAP (*β* ± S.E, − 13.47 ± 2.73; *p*-value > 0.001); moreover, a statistically significant interaction between chronological age and ML-ratio was found (Table 3, model E; *β* ± S.E γ _10*02_, 0.13 ± 0.04; *p*-value < 0.001), meaning that within subjects with the same age, higher ML-ratio levels are directly associated with a larger amplitude of the peroneus-superficialis proximal CMAP. On the contrary, no statistically significant association could be found for the NL-ratio (*β* ± S.E, 0.39 ± 0.29; *p*-value = 0.17, Table 3, model D). In both models, age, sex, their interaction, and multimorbidity were also significantly associated with proximal CMAP.

**Table 3:** Linear mixed model, factors associated to variation of peroneus proximal CMAP according to times of the study (EMG)

			Model A Unconditional means model	Model B Unconditional growth model	Model C Interaction model	Model D Fully adjusted NL-ratio	Model E Fully adjusted ML-ratio
Initial status	Intercept	γ _00_	4.73 ± 0.07***	10.15 ± 0.28***	8.86 ± 0.38***	7.46 ± 0.75***	10.50 ± 0.71***
	Sex (males)	γ _01_			2.62 ± 0.55***	2.58 ± 0.55***	2.75 ± 0.55***
	NL-ratio < 1.96	γ _02 NLr_				0.39 ± 0.29	
	ML-ratio < 0.37	γ _02 MLr_					− 13.47 ± 2.73***
	BMI	γ _03_				0.01 ± 0.01	0.01 ± 0.01
	Multimorbidity	γ _04_				− 0.28 ± 0.04***	− 0.24 ± 0.04***
	CRP	γ _05_				0.01 ± 0.01	0.01 ± 0.01
Rate of change	Intercept^&^	γ _10_		− 0.08 ± 0.01***	− 0.06 ± 0.01***	− 0.04 ± 0.01***	− 0.07 ± 0.01***
	Interaction	γ _10*01_			− 0.03 ± 0.01***	− 0.03 ± 0.01***	− 0.03 ± 0.01***
	Interaction	γ _10*02_				− 0.01 ± 0.01	
	Interaction	γ _10*02_					0.13 ± 0.04 ***
Level 1	Within person	δ ^2^_e_	6.94 ± 0.21***	6.23 ± 0.24***	6.23 ± 0.24***	6.09 ± 0.19***	5.87 ± 0.19***
Level 2	In initial status	δ ^2^_0_	2.54 ± 0.22***	3.26 ± 0.74***	3.13 ± 0.74***	3.11 ± 0.46***	3.30 ± 0.45***
	In rate of change	δ ^2^_1_		0.01 ± 0.09	0.01 ± 0.09	0.10 ± 0.11	0.07 ± 0.11
	Covariance	δ _01_		− 0.29 ± 0.24*	− 0.28 ± 0.24	− 0.25 ± 0.10**	− 0.28 ± 0.08***
		AIC	16,388	15,986	15,961	15,887	15,837

*p < 0.05; **p < 0.01; ***p < 0.001

#### Nerve conduction velocity

In Table 4, we report the linear mixed model analysis for the variation through times of peroneus-NCV. The unconditional means model provides insights into the NCV, independent of individuals and time. The NCV mean value was found to be 43.49 ± 0.10 (*p*-value < 0.001), with within-subjects’ variance of 15.15 ± 0.47 (*p*-value < 0.001) and a variance between subjects of 6.94 ± 0.54 (*p*-value < 0.001). This indicates that 69% of the total variation in the test can be attributed to differences among subjects (Table 4, model A). Moving to the unconditional growth model (Table 4, model B), the age effect was assessed, and it was estimated that distal nerve conduction velocity mean decreases by − 0.08 ± 0.01 (*p* < 0.001) for every year of age. Furthermore, a gender dimorphism was observed, with males exhibiting a higher NCV (*β* ± S.E, 3.09 ± 0.61, *p*-value < 0.001), with more decrease of NCV during the aging process (interaction age for sex, γ _10*01_, − 0.03 ± 0.01; *p*-value < 0.001), compared to females (Table 4, model C). Finally, in the fully adjusted model D, the role of NL-ratio and in model E, the role of ML-ratio were assessed; both models were adjusted for age, sex, their interaction, body mass index, comorbidities score, and markers of low-grade chronic inflammation (CRP). Low ML-ratio levels were associated with a higher mean NCV value (*β* ± S.E, − 11.25 ± 4.16, *p*-value < 0.001, Table 4, model E), independently from all the potential confounders considered. No statistically significant second-order variables could be identified. On the contrary, no statistically significant differences could be found for the NL-ratio (*β* ± S.E, 0.08 ± 0.43; *p*-value = 0.99, Table 4, model D).

**Table 4:** Linear mixed model, factors associated to variation of peroneus nerve conduction velocity according to times of the study (EMG)

			Model A Unconditional means model	Model B Unconditional growth model	Model C Interaction model	Model D Fully adjusted NL-ratio	Model E Fully adjusted ML-ratio
Initial status	Intercept	γ _00_	43.49 ± 0.10***	50.21 ± 0.42***	50.37 ± 0.57***	47.58 ± 1.12***	49.28 ± 1.06***
	Sex (males)	γ _01_			0.09 ± 0.83	− 0.43 ± 0.81	− 0.23 ± 0.81
	NL-ratio < 1.96	γ _02 NLr_				− 0.08 ± 0.43	
	ML-ratio < 0.37	γ _02 MLr_					− 11.25 ± 4.16***
	BMI	γ _03_				0.10 ± 0.02***	0.09 ± 0.02***
	Multimorbidity	γ _04_				− 0.38 ± 0.06***	− 0.31 ± 0.06***
	CRP	γ _05_				− 0.01 ± 0.01	− 0.01 ± 0.01
Rate of change	Intercept^&^	γ _10_		− 0.10 ± 0.01***	− 0.08 ± 0.01***	− 0.07 ± 0.01***	− 0.08 ± 0.01***
	Interaction	γ _10*01_			− 0.03 ± 0.01**	− 0.02 ± 0.01*	− 0.03 ± 0.01*
	Interaction	γ _10*02_				− 0.01 ± 0.01	
	Interaction	γ _10*02_					0.07 ± 0.06
Level 1	Within person	δ ^2^_e_	15.15 ± 0.47***	14.38 ± 0.45***	14.31 ± 0.44***	14.14 ± 0.44***	13.78 ± 0.43***
Level 2	In initial status	δ ^2^_0_	6.94 ± 0.54***	12.30 ± 6.36*	15.16 ± 6.34**	10.60 ± 5.81*	9.92 ± 5.64*
	In rate of change	δ ^2^_1_		0.01 ± 0.004*	0.004 ± 0.0.001**	0.003 ± 0.0.001*	0.003 ± 0.001*
	Covariance	δ _01_		− 0.19 ± 0.11	− 0.24 ± 0.11*	− 0.16 ± 0.10	− 0.14 ± 0.10
		AIC	19,021	18,774	18,618	18,572	18,521

*p < 0.05; **p < 0.01; ***p < 0.001

#### Lower limb muscle power

Over the course of the years (Table 5 γ_10*_γ_10_), lower limb power decreases with an exponential pattern in all three models (model A, *β* ± S.E − 0.05 ± 0.02, *p*-value = 0.008; model B, *β* ± S.E − 0.06 ± 0.02, *p*-value = 0.007; model C, *β* ± S.E − 0.06 ± 0.02; *p*-value = 0.005). Statistically significant higher values of lower limb power are observed in male sex compared to female sex (Table 5, models A, B, C γ_01_). However, during aging, men lose a greater proportion of lower limb power in comparison with women (Table 5 γ_10*_γ_10*_γ_01_). In all three models, changes in body composition (Table 5 γ_04_) and its interaction with quadratic term of age (Table 5 γ_10*_γ_10*_γ_04_) show a direct correlation with lower limb power. The proximal CMAP (Table 5, model A *β* ± S.E γ_03P_, − 1.15 ± 0.24), distal CMAP (Table 5, model B *β* ± S.E γ_03D_, − 0.96 ± 0.21), and NCV (Table 5, model C *β* ± S.E γ_03NCV_, − 0.43 ± 0.16) were all indirectly statistically significantly associated with lower limb power; no higher order interaction is found. Considering ML-ratio interaction with age, the association with lower limb power was direct (Table 5, models A, B, C γ_10*_γ_02_). Finally, multimorbidity is indirectly associated with changes in lower limb power.

**Table 5:** Linear mixed model, factors associated to variation of lower limb muscle power according to times of the study and markers of (EMG). Model A, proximal CMAP; model B, distal CMAP; model C, nerve conduction velocity

		Model A	*p*-value	Model B	*p*-value	Model C	*p*-value
		*β* ± S.E		*β* ± S.E		*β* ± S.E	
Intercept	γ_00_	− 46.71 ± 73.10	0.52	− 52.47 ± 73.17	0.47	− 43.69 ± 73.12	0.55
Male sex	γ_01_	62.53 ± 25.89	0.02	63.10 ± 25.93	0.02	62.85 ± 25.86	0.02
ML-ratio	γ_02_	− 145.94 ± 38.44	< 0.001	− 138.49 ± 38.42	< 0.001	− 139.05 ± 38.32	< 0.001
Proximal CMAP	γ_03P_	− 1.15 ± 0.24	< 0.001				
Distal CMAP	γ_03D_			− 0.96 ± 0.21	< 0.001		
NCV	γ_03NCV_					− 0.43 ± 0.16	0.007
Body mass index	γ_04_	14.16 ± 2.89	< 0.001	14.29 ± 2.90	< 0.001	14.38 ± 2.89	< 0.001
Multimorbidity	γ_05_	− 3.51 ± 0.64	< 0.001	− 3.49 ± 0.64	< 0.001	− 3.32 ± 0.64	< 0.001
C-reactive protein	γ_06_	− 0.14 ± 0.10	0.15	− 0.15 ± 0.10	0.13	− 0.16 ± 0.10	0.11
Chronological age	γ_10_	5.75 ± 2.55	0.02	5.87 ± 2.56	0.02	6.14 ± 2.55	0.02
Chronological age * male sex	γ_10*_γ_01_	3.20 ± 0.87	< 0.001	3.17 ± 0.87	< 0.001	3.07 ± 0.87	< 0.001
Chronological Age *ML-ratio	γ_10*_γ_02_	1.68 ± 0.52	< 0.001	1.59 ± 0.52	0.002	1.61 ± 0.52	0.002
Chronological age * body mass index	γ_10*_γ_04_	− 0.37 ± 0.10	< 0.001	− 0.37 ± 0.10	< 0.001	− 0.38 ± 0.10	< 0.001
Chronological age ^2^	γ_10*_γ_10_	− 0.05 ± 0.02	0.008	− 0.06 ± 0.02	0.007	− 0.06 ± 0.02	0.005
Chronological age ^2^* male sex	γ_10*_γ_10*_γ_01_	− 0.04 ± 0.01	< 0.001	− 0.04 ± 0.01	< 0.001	− 0.04 ± 0.0	< 0.001
Chronological age ^2^* body mass index	γ_10*_γ_10*_γ_04_	0.002 ± 0.001	0.002	0.003 ± 0.001	0.002	0.002 ± 0.001	0.001

## Discussion

In the InCHIANTI study population, subjects with lower values of ML-ratio have higher conduction velocity and higher proximal and distal action potential values, but taking into account the interaction between age for ML-ratio effect, subjects of the same age with higher ML-ratio show better EMG markers. Moreover, age, sex, multimorbidity, body mass index, electrophysiological parameters, and ML-ratio predict lower limb power variation (powerpenia) across the time of the study. Specifically, lower limb muscle power is indirectly associated with ML-ratio, but again considering the interaction between age for ML-ratio effect, subjects at the same age with higher ML-ratio showed higher muscle-power, in all the three models that consider proximal CMAP, distal CMAP, and nerve conduction velocity, respectively. Male subjects showed higher lower limb power, but during aging, they suffer greater loss compared to females. Finally, an indirect correlation is observed between the change in proximal CMAP, distal CMAP, and in NCV and lower limb muscle-power.

Several circulating innate immune cell subsets, including macrophages, monocytes, lymphocytes, and neutrophils, contribute to the pathogenesis or take part in the progression of different diseases and more in general also in the aging processes. In the Baltimore Longitudinal Study of Aging (BLSA), the absolute neutrophil count and the derived NL-ratio were predictors of all-cause mortality and multimorbidity, whereas the absolute lymphocyte count did not result in having a role [33]. Further, in the Mugello study, a representative Italian cohort of free-living nonagenarians, we demonstrated that subjects affected by dementia had a higher lymphocyte count and lymphocyte-to-monocyte ratio compared to the non-demented nonagenarians [21]. In the Maastricht Study, an observational prospective population-based cohort that is characterized by an oversample of patients with type 2 diabetes, higher absolute number of basophils, and higher percentage of CD4 + T cells were cross-sectionally associated with a reduced NCV, whereas CD8 + T cell percentage pointed to the opposite. These findings implicate that both innate immunity and adaptive immunity are involved in the nerve myelin dysfunction in large fiber nerves [34]. The authors stated that it was unclear to “what extent this observation would be relevant” in the contest of pathophysiology of polyneuropathy. They also suggested that a high proinflammatory state was linked to polyneuropathy [35]. In a cross-sectional Turkish electrophysiology outpatient population study, diabetic patients with a reduced CMAP amplitude showed higher values of monocyte-to-HDL cholesterol ratio (an indicator of inflammation and oxidative stress) compared to non-diabetic and to non-neuropathic patients [36], suggesting that inflammation and oxidative stress modulate the pathogenesis of polyneuropathy.

In line with these studies, in the InCHIANTI study, we found an inverse association between the age-related variation of the ML-ratio and the NCV, and proximal and distal CMAP variation. Interestingly, when we have considered in the models the interaction between chronological age and the ML-ratio, this association changes in direction, i.e., for patients with the same chronological age, ML-ratio was directly associated with higher EMG-derived parameters. Meaning that with aging, more efficient peripheric nervous system can be found in those individuals with a more effective balance between innate and adaptive immunity. Lastly, the change in the association direction between EMG-derived parameters and the ratio, when the age effect is considered, could at least partially explain why the role of monocyte infiltration in the nervous system degeneration is controversial, with some authors reporting higher monocyte number and others finding a reduction [37]. As previously described in the InCHIANTI study, a sexual dimorphism was observed in the trend of EMG markers [14, 15]. In our analysis, male sex shows higher mean values in proximal and distal CMAP, and in NCV, but while aging, male subjects have a more pronounced reduction in all the three EMG markers, compared to females.

In our study, lower limb power decreases during aging with a curvilinear function [2, 38]; it shows a gender dimorphism, and specifically male subjects reach a higher peak in youth and muscle power decrease at a progressively increasing rate during aging [38, 39]. Moreover, changes in lower limb muscle power are directly correlated with age-related changes in body composition, and this is not gender related. This is partially in contrast with the results of the Copenhagen Sarcopenia Study, where the age-related variation in the body mass showed a different trend according to gender, and appeared to contribute to the age-related decrease in lower limb power [38]. These contrasting results between the two populations may be due to at least two important reasons: the first inherent in the study design, longitudinal with repeated measures in the InCHIANTI, and cross-sectional in the Copenhagen Sarcopenia Study; consequently, the statistical analysis approach is substantially different, affecting also inference. In addition, our analysis approach also included the study of the role of immune and neurological resilience in the variation of lower limb power, whereas the Copenhagen Sarcopenia Study had as endpoint to provide and describe only the epidemiological trend in lower limb variation. Moreover, in our analysis to assess the role of muscle fat infiltration as a potential effect modifier in lower limb power [40], we have considered in our models: muscle fat infiltration and subcutaneous fat measured via pQCT, but we did not find a statistically significant association, and the fit of the models did not change; therefore, we do not include those terms in our results.

Existing evidence supports the role of several diseases and multimorbidity in the pathway leading to muscle aging and affecting muscle mass, density, and strength [2, 22, 33]. Therefore, it is not surprising that also power reduction (powerpenia) could be affected by multimorbidity. Probably, for diseases induced reduction of physical activity or, in the case of neurological diseases, for a direct action on muscle [7]. A support for an action in lower limb power reduction of the nervous system can also be inferred from our data, where the three EMG components—distal CMAP, proximal CMAP, and NCV—are all indirectly related to powerpenia independently of the other confounders considered. To explain the apparently surprising result (indirect association between distal CMAP, proximal CMAP, and NCV and lower limb power strength), several hypotheses might be advanced. The idea that an impairment of the membrane electrical processes results in an enlargement in the compound muscle action potential is counterintuitive, but it was theoretically hypothesized and also demonstrated, with an impaired sarcolemma membrane excitability, as found during aging, manifested by an increase of compound muscle action potential size [41]. Moreover, during brief but high-intensity contractions (as for the assessment of lower limb power), a second phase of the compound muscle action potential of greater amplitude and shorter duration than that evoked during slight contractions occurs [41, 42]. However, the lack of any potential interaction between distal CMAP, proximal CMAP, and NCV and other confounders included in the analysis does not exclude that the indirect association could be biased by some other confounders not considered in our models.

During aging, a chronic low-grade inflammation often referred to as inflammaging [43] was reported to be associated with components of the aging muscle [44], but also with central nervous system diseases [45]. The contribution of the peripheral immune system to neurodegeneration or the etiopathogenesis of neurological diseases is far from being clarified. Monocytes and macrophages share myeloid origin, even if they follow different development and maturation paths; moreover, they both show phagocytic activities and can release toxic and chemotactic factors [37]. With the loss of axonal integrity, Schwann cells trigger Wallerian degeneration and initiate a chemotactic action that involves leukocyte infiltration. Monocytes and macrophages actively start myelin debris removal, a key step for axonal reparative processes [46]. At least three mechanisms by which peripheral immune cells interact with CNS can be hypothesized: (1) direct infiltration of immune cells in the CNS through the damaged blood–brain barrier [37, 47]; (2) by immune cells releasing molecules that act locally, but could also access backward the central nervous system [37, 48]; (3) activation of monocytes/macrophages that interact with injured peripheral axons to modify the response of entire motoneurons and consequently modify the activation of microglial cells [37]. The CNS damage causes a reduction in the numerosity of type IIA muscle fibers, and such fibers are more involved in power production compared to type I fibers [40]. Moreover, the loss of motoneurons induces a reduction in the firing rates to produce fused contractions and impaired neuromuscular activation with a reduction in muscle power [49, 50]. The hypothesis of a neuro-immune interaction in the pathway leading to muscle aging and affecting muscle mass, density, and strength is somehow confirmed in an experimental injury model, where a transection anastomosis model of the sciatic nerve is employed [51]. In the experimental group at the suture site was given peripheral blood mononuclear cells (PBMCs). In the experimental group, PBMCs improved nerve regeneration, weight rate, and muscle fiber structure, probably through the modulation of the complement and the coagulation cascade pathways [51].

### Limitations

The main limitation of the present study is the so-called crosstalk, artifacts related to co-stimulated muscles that could alter the results of ENMG. However, this must be considered as a systematic error, since all the examinations were performed by the same operator, with the same equipment, and with the same protocol, in all the study times. Always related to instrumental results, another potential bias to consider is the ceiling effect of markers such as lower limb power or NCV and CMAP. This is an age-related phenomenon and therefore implicit in all epidemiologic studies of aging. Lastly, we used a proxy measure to assess IR, the ratios between innate and adaptive immunity. However, there is a lack of consensus to define IR, and only recently the SPRING study is trying to specifically elucidate the dynamics of stress response systems [52]. We were obviously unable to assess the immunological response when a new disease or a change in function occurred, due to the epidemiological study design. Therefore, we modeled the allostatic load (i.e., the onset of new diseases) and immunocompetence (i.e., changes in the ratios) as continuous time-dependent functions.

Among the strengths of this study, we must consider that the InCHIANTI study is population-based and representative of the age and sex of the free-living Italian population. Moreover, the long follow-up study (9 years) allows us to observe the trajectory of the aging processes. Finally, the possibility of taking into account, in a multivariate analysis, several potential confounders that were accurately and objectively measured in all members of the cohort [53].

## Conclusion

Although these findings do not provide an indication of the specific neural mechanism causing a deficit in lower limb muscle power, they do suggest that the decline in performance of the peripheral nervous systems during aging is closely linked to the age-related decrease in muscle function and the phenomenon of chronic low-grade age-related inflammation. Our results may support the hypothesis that powerpenia shares etiopathological moments with dynapenia, but at the same time may represent a different nosological entity, allowing tailored interventions of physical activity in the prevention of the aging muscle loss of function.

## Supplementary Information

Below is the link to the electronic supplementary material.

Supplementary file1 (DOCX 108 KB)

Supplementary file2 (DOCX 432 KB)

## Data Availability

The datasets used and/or analyzed during the current study are available from the responsible authors for the InCHIANTI study (Luigi Ferrucci) on reasonable request. Data of the InCHIANTI study is available to all researchers upon justified request using the proposal form available on the InCHIANTI website (https://www.nia.nih.gov/inchianti-study, accessed on 04/13/2023).

## Declarations

### Patient and public involvement

Patients and/or the public were not involved in the design, conduct, reporting, or dissemination plans of this research.
