# Supplementary material for: Role for neurological and immunological resilience in the pathway of the aging muscle powerpenia: InCHIANTI study longitudinal results
Source: GeroScience. 2025 Jan 30;47(4):5591–604. doi: 10.1007/s11357-025-01536-6 (PMC12397046; doi:10.1007/s11357-025-01536-6)
Supplement: Supplementary file 1 — Supplementary file1 (DOCX 108 KB) [file 11357_2025_1536_MOESM1_ESM.docx]

Supplementary Figure 1. Methodological standardization of the Surface electroneurography in the InCHIANTI-Study.

| 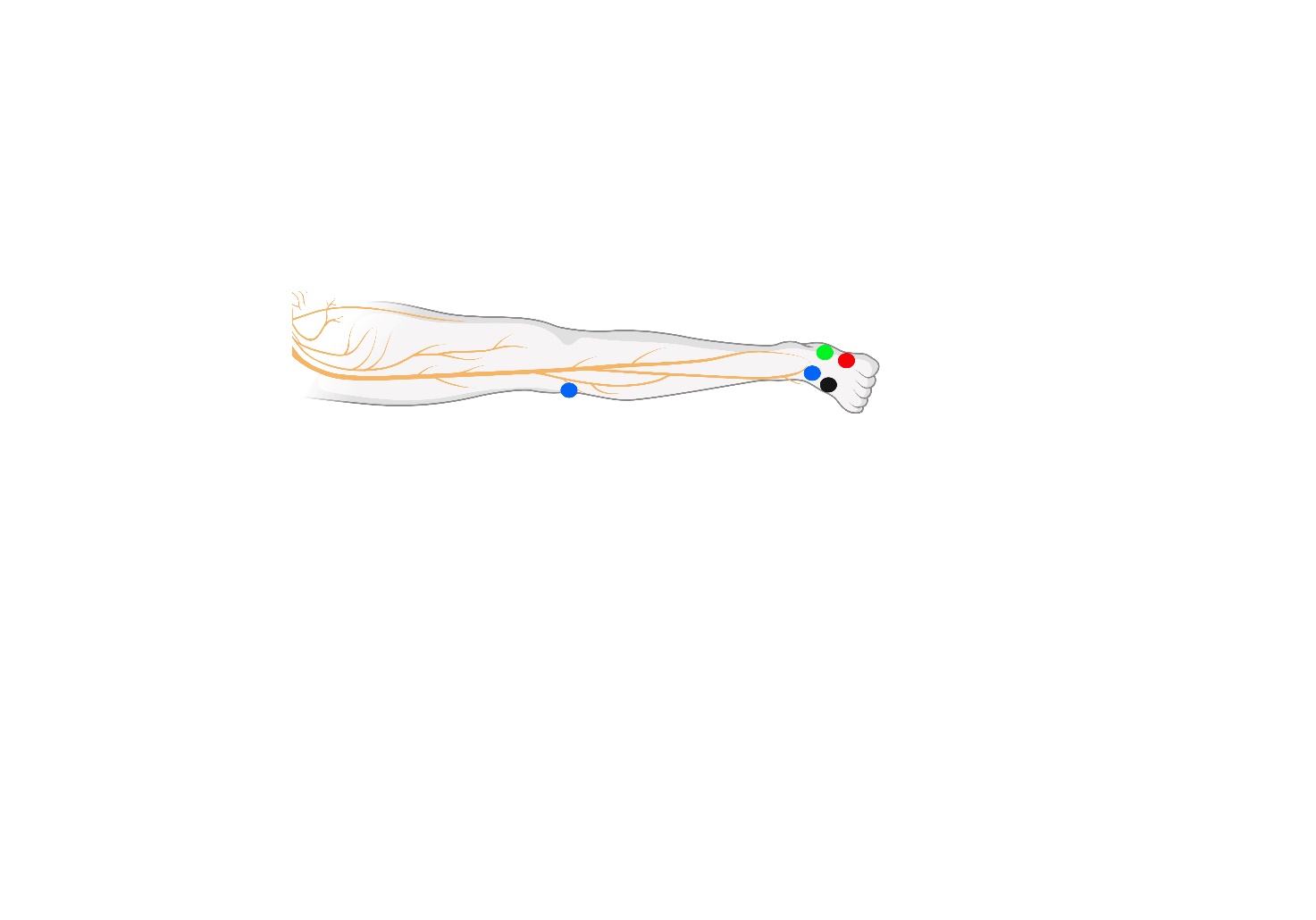 |
| --- |

According to Kimura ^1^, a disposable surface recording electrode was placed on the dorsum of the foot over the belly of the extensor digitorum brevis (black circle); the peroneal nerve was stimulated with a bipolar electrode, with distal stimulation applied approximately 8 cm proximal to the recording, just lateral to the tibialis anterior tendon (blue circle). More proximal, the nerve was stimulated just below the head of the fibula (blue circle). A ground electrode was placed between the stimulating and recording electrodes (green circle). Stimulation began with a very mild electrical pulse to check that the electrode position was appropriate. The stimulus was then gradually increased until the recorded muscle response (a sinusoidal wave) reached its maximum amplitude.

The following parameters of nerve conduction studies were measured: (a) the amplitude of the compound muscle action potential (CMAP), which is related to the number of axons conducting impulses from the stimulus point to the muscle and the number of functioning motor endplates. It was measured from peak to peak of the action potential. (b) Nerve conduction velocity (NCV), calculated by dividing the length of the nerve segment between the two stimulation points by the difference between the proximal and distal time latencies, which reflects the conduction velocity of the fastest motor axons. Electrical averaging was performed periodically during the test measurement to reduce the signal-to-noise ratio. According to literature electromyograph setting were: frequency 8 Hz to 8 kHz; sweep speed 5 ms/div; gain 1.000 μV.

1. Kimura J. Assessment of Individual Nerves. Electrodiagnosis in Diseases of Nerve and Muscle: Principles and Practice. 3rd Edition. Oxford University Press, editor. New York; 2001.
