# Supplementary material for: Role for neurological and immunological resilience in the pathway of the aging muscle powerpenia: InCHIANTI study longitudinal results
Source: GeroScience. 2025 Jan 30;47(4):5591–604. doi: 10.1007/s11357-025-01536-6 (PMC12397046; doi:10.1007/s11357-025-01536-6)
Supplement: Supplementary file 2 — Supplementary file2 (DOCX 432 KB) [file 11357_2025_1536_MOESM2_ESM.docx]

***Supplementary Figure 2: Lower limb muscle power evaluation***


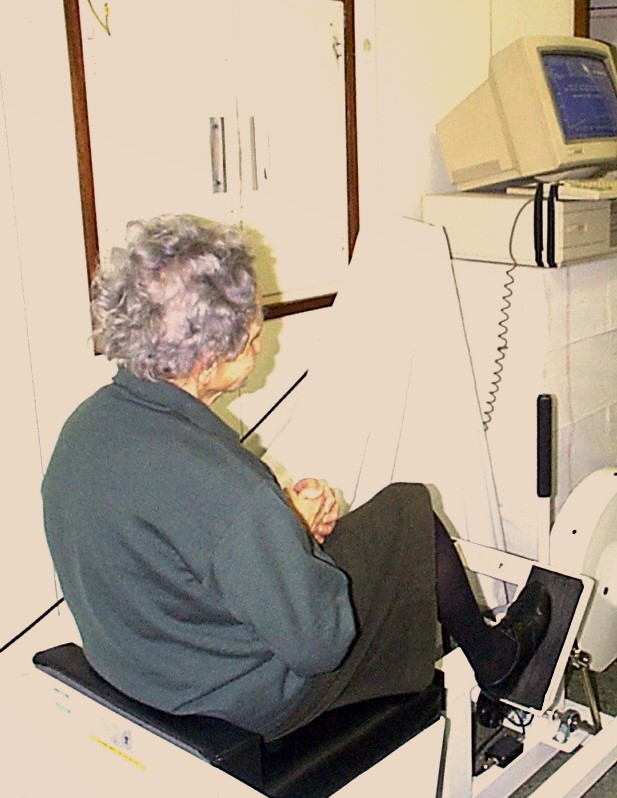


The main purpose of the leg extensor power rig is to provide a safe and convenient method of assessing power output from the lower limb. The average power output from a subject is derived from the final velocity of the flywheel following an explosive push from the subject in the leg extensor rig. The rotation of the flywheel is recorded using an optical switch on the rig, which is connected to a digital interface board and a computer/laptop via a USB cable. The Leg Rig analysis software provides the means to display, record and compare performance and calculate explosive power.

**Calibration Factors:** These depend on the inertia of the flywheel and the force of the return spring. Once established they do not need to be checked.

**Clothing:** There is no need for special clothes, but tight skirts, tight jeans and high heeled shoes should be avoided.

**Setting up:** The seat clamps are released, and the seat is pushed towards the flywheel, leaving space for the subject. The chair is rotated so that the subject can easily sit down and place their feet on the footrest. With the subject facing the flywheel, the chair is swung into line with the skids. The subject places one foot on the foot pedal. The choice of foot depends on the observer but should always be consistent within a study (dominant or right or left). The subject gently presses the foot pedal all the way down and then continues to press, slowly moving the seat back until the leg is almost fully extended. The observer then tightens the seat and checks that the subject can achieve full leg extension by compressing the back cushion without rising in the seat. The sitting position can be recorded using the tape measure attached to the chair.

**Measurement**

Body position: The subject keeps the arms folded and the inactive leg relaxed on the floor. The active leg rests on the push-pedal with the heel resting against the lower and inner lips.

**Practice push:** The subject leans forward slightly and pushes the pedal submaximal all the way down to full extension and then slowly allows the pedal to return to the start position under the influence of the return spring. The whole sole of the foot, including the heel, should remain in contact with the pedal all the time.

**Performance:** The subject pushes the pedal down as hard and fast as possible. Strong verbal encouragement is given by the observer. Feedback from the display can be allowed at the observer’s discretion. The pedal is returned slowly to its start position, the flywheel is then braked and reset. The subject then tries again. Rest periods of about 30 secs. are sufficient and up to 9 efforts may be needed to reach a convincing maximum power output.

**Precautions**

Before adjusting the starting position by hand, the flywheel should be braked with extreme care.

It is important to avoid any risk of uncontrolled return of the pedal, so the subject should let it rise slowly to its starting point before removing their foot.

The subject should not throw the upper body backwards against the back of the chair during the push.

Elderly or frail subjects can be supported in the seat by an observer until they are ready to make their push.
